# Infective endocarditis by HACEK: a review

**DOI:** 10.1186/s13019-022-01932-5

**Published:** 2022-08-19

**Authors:** Mansoor Khaledi, Fatemeh Sameni, Hamed Afkhami, Jaber Hemmati, Aram Asareh Zadegan Dezfuli, Mohammad-Javad Sanae, Majid Validi

**Affiliations:** 1 Department of Microbiology, Faculty of Medicine, Shahed University, Tehran, Iran; 2 Department of Microbiology, Faculty of Medicine, Hamadan University of Medical Sciences, Hamadan, Iran; 3 Department of Microbiology, Faculty of Medicine, Ahvaz Jundishapur University of Medical Sciences, Ahvaz, Iran; 4 Cellular and Molecular Research Center, Basic Health Sciences Institute, Shahrekord University of Medical Sciences, Shahrekord, Iran

**Keywords:** Endocarditis, Congenital heart disease, HACEK, Echocardiography

## Abstract

Infective endocarditis (IE) is a severe disease that is still associated with high mortality despite recent advances in diagnosis and treatment. HACEK organisms (*Haemophilus *spp., *Aggregatibacter actinomycetemcomitans*, *Cardiobacterium hominis*, *Eikenella corrodens*, and *Kingella kingae*) are gram-negative bacteria that are part of the normal flora of the mouth and upper respiratory tract in humans. These organisms cause a wide range of infections, of which IE is one of the most notable. In order to control and prevent endocarditis caused by HACEK, measures such as oral hygiene and the use of prophylactic drugs should be used for people at risk, including people with underlying heart disease and people with artificial valves. This review is a summary of the main aspects of IE focusing on HACEK organisms.

## Background

Infective endocarditis (IE) is caused by infection of the heart valve blood by fungi and bacteria (this bacteremia can cause septic symptoms) and their vegetative growth, which consists mainly of platelets, microorganisms and fibrins. The superficial growth can cause embolism in many organs such as the kidneys, lungs, skin, brain, and central nervous system, leading to signs of divergence. If the patient with infective endocarditis does not receive adequate treatment, the disease can be lethal [1]. Diagnosis of IE is still difficult, although blood culturing methods, accurate diagnostic criteria and echocardiography have improved [2, 3]. Clinician may also notice possible illnesses such as stroke, heart failure, pulmonary embolism, meningitis, nephritis, collagenosis, pneumonia, or urinary tract infections due to the variety of symptoms in this disease [1].

## Main text

### Epidemiology

Recently, there have been significant changes in the epidemiology of IE based on the pathogens involved in the disease [4]. Infectious endocarditis is a rare disease with an annual incidence of 3 to 10 cases per 100,000 people [5]. The disease is reported to be twice as common in men as in women. Today, the average age of patients with infective endocarditis is over 65 years, which is probably due to the increasing prevalence of predisposing factors such as artificial valves, acquired valve disease, diabetes mellitus and hemodialysis in this group of people [6].

IE mortality has varied between 13 and 25% and it has been observed that 9–20% of most patients die within the first year after discharge [7]. IE mortality rate in Africa is reported to be more than 26% [8]. In developed countries, the annual prevalence of IE is estimated at 3–9 patients per 100,000 people, an increase that has been seen since 1970–2013 [9]. Despite improvements in patients' prevention and surgery methods, improved antibiotic therapy, and widespread advances in imaging technology, the mortality rate is now high [10]. In developed countries, the epidemiology of IE has changed dramatically, with the number of IE patients as well as the rate of PVE gradually increasing over the last two decades, with staphylococci being the most significant pathogen involved in IE [11]. The prevalence of IE in Germany between 2005 until 2015 was 11.6 per 100,000 people, which has increased over the years under study [12].

The epidemiology of the disease varied in developing countries, with streptococci being the most significant pathogens involved, Rheumatic heart disease (RHD) and Congenital Heart Disease (CHD) being the most common underlying heart disease, and the number of patients receiving surgical treatment increasing [13, 14].

### IE In children

Infectious endocarditis is a life-threatening disease in children's communities. The disease is associated with high mortality [15]. According to some studies, the prevalence of infectious endocarditis in children was lower than in adults, and the rate of hospitalization in children was lower. The prevalence of infective endocarditis is higher in children with CHD and underlying rheumatic disease [16].

Approximately 35–60% of IE infections have CHD, which is a risk factor for IE in children. Congenital heart disease is one of the most common causes of IE in high-income countries. The clinical features of the disease in children vary, which can include weight loss, fever, heart damage, or septic embolization [17, 18].

Recently, an increase in IE cases in children has been reported, which has been observed in these patients with structural heart problems, especially after surgical repair of lesions. More than half of children with IE have children with CHD who have a history of heart surgery. The use of a central venous catheter increases the risk of IE even in children without CHD. It should be noted, however, that a study reported that more than 10% of IE children had no history of any risk factors for the disease, including CHD [19].

The most common IE pathogens in children are gram-positive cocci, especially virulence group α-hemolytic streptococci, staphylococci and enterococci. The HACEK group is one of the rare causes of IE, which accounts for 1.4% of endocarditis cases [20].

The prevalence of infectious endocarditis in children between 1972 and 1982 was 0.75 per 1000 hospitalized [16]. It is noteworthy that between 2003 and 2010, the admission rate of infectious endocarditis per 1000 patients was reported between 0.05 and 0.12 [20, 21].

The annual prevalence of IE is reported to be 0.05–0.12 cases per 1000 hospitalized children and the resulting mortality rate is about 5% [22, 23]. According to studies, the annual prevalence of IE in children in the USA was between 0.34 and 0.64 cases per 100,000 people and approximately 0.05–0.12 people per 1000 children are hospitalized [19].

### Molecular genetic methods and histology in valvular culture

**Fig. 1:**
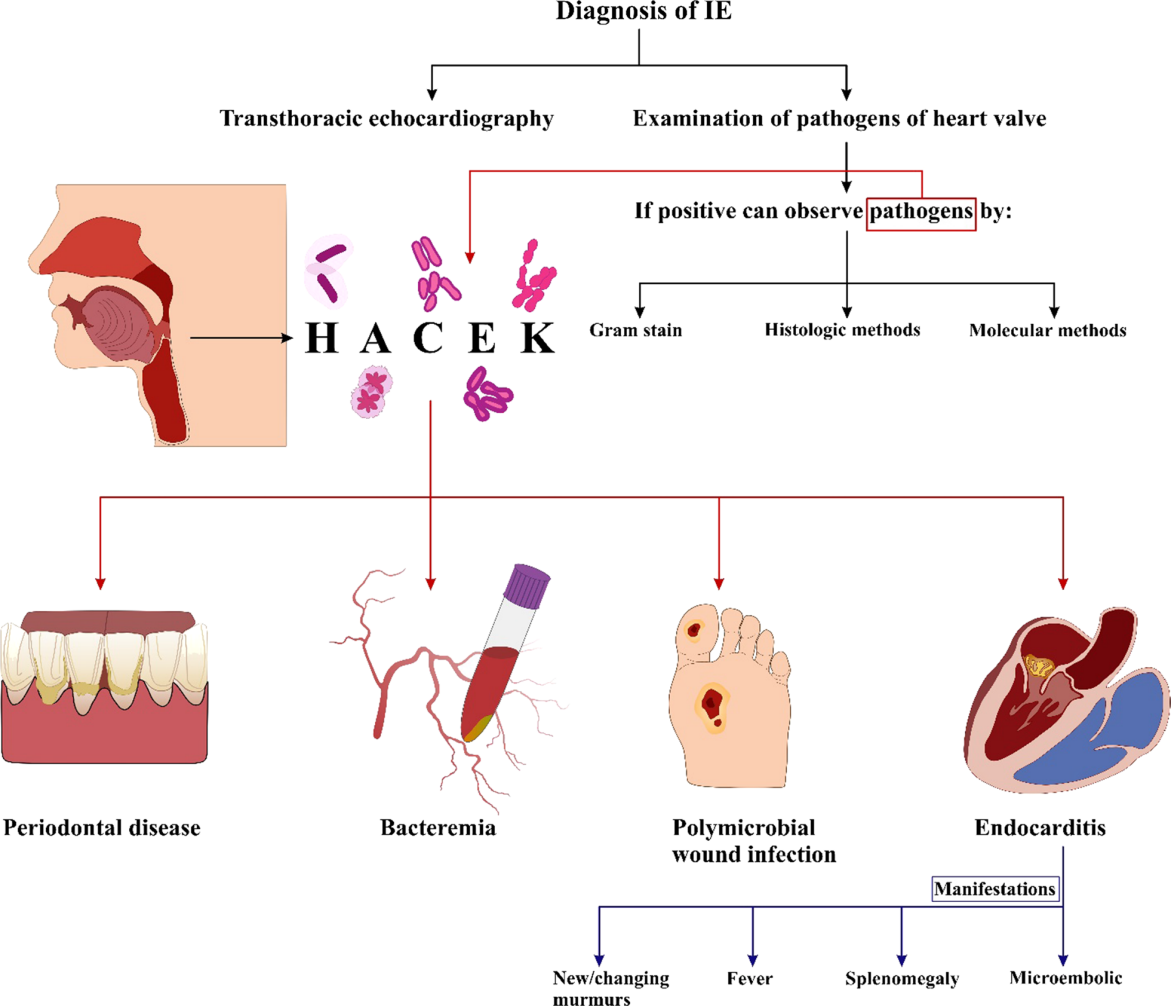
Diagnosis of IE

Examination of the heart valve is the gold standard for diagnosing the IE [1, 3, 24]. If positive, we can identify pathogens in biopsy of the valves from an IE patient by using culture including Gram stain [25]. Histological methods include histochemistry [26] or molecular genetic methods such as PCR [27–29] (Fig. 1). Due to the high concentration of microorganisms in plants, these methods can be used and most of them can be successfully identified [1] by the ways, valvular culture has low negative predictive value for detecting the microorganisms (56%) [28] because of its low sensitivity (15%) [30], (13%) [28]], and it is not high specified [31]. However, due to the contamination of the heart valve, we may also see a false positive result in the sample cut from the heart valve during surgery [31]. Sometimes microorganisms detected by methods such as histopathology or PCR from the heart valve may remain there for months after the IE patient has recovered; Therefore, in addition to the experimental methods used, we must ensure the clinical diagnosis of the presence or absence of IE in the patient [25, 32–34].

### Echocardiography

Echocardiography is a non-surgical test that can show moving images of a patient's heart with the help of sound waves. The displayed images include the size, shape and quality of how heart valves and chambers working. Echo can detect possible blood clots inside the heart, problems with the aorta and the fluid buildup in the pericardium (the sac around the heart). It is also used to detect heart problems in infants and children [35].

Transthoracic echocardiography (TTE) based on sound transmission through the thoracic wall may allow visualization of vegetations and heart function. The subsequent development of transesophageal echocardiography (TEE) with the sound-emitting probe in the esophagus closer to the heart improved such transmissions substantially. With its higher sensitivity TEE detects small vegetations, infection of prosthetic heart valves, infected pacemaker leads and perivalvular abscesses. However, TTE has improved with the second harmonic imaging [1, 36–38].

### Infective Endocarditis caused by hacek microorganisms

#### Endocarditis caused by HACEK organisms

HACEK endocarditis mostly affects most patients with heart disease or artificial valves, and is characterized by an insidious course, with a mean diagnosis delay of 1 month (*Haemophilus spp.*) to 3 months (*Aggregatibacter* and *Cardiobacterium spp.*) [39]. In Olmsted County, Minnesota, about 0.14 of every 100,000 people develop HACEK endocarditis each year. 12 cases occurred in patients with prosthetic valve, and in patients with native valve, 33 casses occurred [40].

The HACEK group of bacteria—*Haemophilus parainfluenzae, Aggregatibacter spp. (A. actinomycetemcomitans, A. aphrophilus, A. paraphrophilus,* and *A. segnis), Cardiobacterium hominis and valvarum, Eikenella corrodens, Kingella kingae and denitrificans*- are fastidious gram-negative bacteria, part of the normal microbiota of oral and upper respiratory tract in humans. Although their pathogenicity is low, they are responsible for 1–3% of infectious endocarditis [39].

Bacteria of the HACEK group are grouped together in infectious endocarditis due to similar characteristics such as: presence in the pharyngeal microbiota, low virulence, similar infections and most importantly (IE) [41]. Hard-growing Gram-negative organisms of primary oral origin are the main cause of infectious endocarditis [39]. Common features of HACEK microorganisms are that they grow slowly and often colonize the oropharynx, and if we add CO_2_ in their medium culture, their growth will be enhanced [40].

HACEK microorganisms cause disease such as periodontal infections, bacteremia, endocarditis, and polymicrobial wound infections [36] otitis media, and abscesses [36, 42, 43]. Native-valve or prosthetic-valve endocarditis caused by HACEK microorganisms is associated with a favorable prognosis [44, 45].

Cases of endocarditis caused by HACEK microorganisms were studied, the etiologic agent for *Actinobacillus actinomycetemcomitans* was 9 patients (20%). The etiologic agents for other microorganisms of the HACEK group were as follows: *Eickenella corrodens*, 2 patients (4%); *Haemophilus aphrophilus*, 7 patients (16%); *Haemophilus parainfluenzae*, 12 patients (27%) *Cardiobacterium hominus*, 12 patients (27%); and *Kingella kingii*, 3 patients (7%) [40]. This study shows the highest and lowest causes of endocarditis, respectively is *Actinobacillus actinomycetemcomitans* and *Eickenella corrodens.*

The demographic data on patients with HACEK endocarditis)the most prevalent agents were Haemophilus parainfluenzae, Haemophilus aphrophilus, and Actinobacillus actinomycetemcomitans, Cardiobacterium hominus, Eickenella corrodens, and Kingella kingii, respectively) shows that most patients, presented with fever, and most patients presented with splenomegaly, new or altered murmurs, and micro embolic or immunologic phenomena. Approximately half of the patients had heart disease, including bicuspid aortic valve, coarctation of the aorta with aortic insufficiency associated with Marfans syndrome, mitral valve prolapse, atrial and ventricular septal defects, or valvular heart disease associated with preexisting rheumatic fever [40].

The pathogenesis of HACEK endocarditis is thought to be due to colonization of the oropharynx with bacteria that reach the vascular space following either trauma or local infection [46–49].

The most positive predictive value (PPV) of bacteremia for endocarditis varied with HACEK species, ranging from 100% for *A. actinomycetemcomitans* to 0% for *E. corrodens*. *A. actinomycetemcomitans* in blood cultures is always associated with endocarditis [50] and there is just a low percentage sometimes shows pneumonia or periauricular osteoradionecrosis in the setting of nasopharyngeal carcinoma [50, 51].

Actinobacillus actinomycetemcomitans, is a part of the natural flora of the mouth and it is often found in human periodontal culture, it is an important pathogen that causes a variety of invasive infections, especially infective endocarditis [51].

A number of patients with invasive infections due to *A. actinomycetemcomitans*, had about 50% previously reported cases, that most of them had *A. actinomycetemcomitans* endocarditis, and there were very few with osteonecrosis,and pneumonia and chest wall mass lesion [51–53].

## Results of the proposed pathogenesis studies of HACEK endocarditis

1. The microorganisms are located in the oropharynx [40, 54] and enter the vascular chamber at the time of dental work or in the context of periodontal disease [55–57], almost half of the patients had either weak teeth or recent dental work within the study [40].
2. The microorganisms are generally considered to be of low virulence and infect structurally damaged or prosthetic cardiac valves, they mostly occur in patients that have preexisting structural cardiac disease, also there is a medium amount in patients with prosthetic valves [40].
3. These microorganisms are fastidious and slow growing, also they may cause infection for a prolonged period prior to diagnosis [27, 40, 54, 58].
4. Various studies have shown that these microorganisms have low growth or no growth in blood culture and this may lead to delay in microbiological diagnosis in patients with negative blood culture. HACEK should be considered in the differential diagnosis of negative endocarditis culture [40, 45].
5. These microorganisms have historically been susceptible to β-lactam antimicrobials [59] despite reports of beta-lactamase production [60].
6. The diagnosis of HACEK endocarditis of either native or prosthetic valves is associated with a good prognosis [44, 45].

### Diagnostic measures

The HACEK group are known as blood culture-negative organisms but if organisms are exposed to carbon dioxide or enriched blood culture medium, they are more easily identified [40, 61, 62]. Better results on grow of these microorganisms is by subculture in 5% sheep blood and chocolate agar at 37° C under 5–10% CO2 [27, 63]. In addition, the use of BacT/Alert system in the first 5 days of blood culture is 99% sensitive in identifying the organism [64]. According to the European IE Guidelines for 2009, long-term incubation is recommended only if IE detection of negative cultures after 72.Hr [65]. prolonged incubation has no value in diagnosing culture negative endocarditis [66].Contrary to previous notions about culturing HACEK organisms, which are very difficult, experience has shown that this group separates after an average incubation period of 3 days, with an average of 4.3 days when using automated blood culture systems [62, 67]. Additionally, it is not true that longer incubation is more effective in the growth of HACEK organisms [68].American Heart Association (AHA) asserts that Initially negative cultures are involved in all patients with suspected IE for more than 2 weeks [69]. Recently, Laser ionization by matrix-time flight mass spectrometry (MALDI-TOF MS) has been very successful in identifying highly sensitive HACEK organisms. It has also been shown that MALDITOF MS allows the earlier identification of species belonging to the HACEK group. [70]. When the HACEK organism grows on a blood culture (under the conditions above), gradient tests, such as E-test (strip impregnated with antimicrobials), are used to determine the susceptibility of the organism to different antibiotics [71].

### Treatments

Delay in diagnosis and its complications can be fatal for humans. Ampicillin in combination with gentamicin [62] is standard treatment for 4 weeks for normal valve infection and for 6 weeks for synthetic valve infection. Surgical procedures follow the recommendations of the Endocarditis Working Group of the International Society of Chemotherapy [72].

The guidelines recommend that ampicillin-resistant organisms be considered because of the difficulty in detecting susceptibility and the potential for resistance among HACEK microorganisms. Therefore, broad-spectrum cephalosporins or fluoroquinolones are considered the first line of treatment for HE (HACEK endocarditis) [73, 74].

Ampicillin should not be prescribed to patients with HE unless the organism is found to be sensitive, and gentamicin is no longer recommended due to its nephrotoxic risks [62, 69]. The standard treatment for HE is intravenous ceftriaxone monotherapy with 2 g per day. The diet should be given for 4 weeks in NVE (native valve endocarditis) and for 6 weeks in prosthetic valve endocarditis [69, 73]. Alternative, rarely used options in cases of β- lactam allergy include fluoroquinolones such as levofloxacin, ciprofloxacin or moxifloxacin [73, 74].

One of the benefits of surgical intervention is valve tissue resection, which can be used for microbiological documentation [58]. However, surgical intervention is necessary in case of heart failure, paravular proliferation, persistence of infection despite appropriate antimicrobial treatment and/or prevention of embolism. These indications for surgery apply to native, as well as prosthetic, valve HE [73, 75]. After surgical treatment, If tissue cultures are negative, antibiotic treatment should be continued to complete the preoperative period, starting with the first negative blood culture after diagnosis of HE. In case of positive tissue cultures, a full course of antibiotics should be started from the time of surgery according to the sensitivity characteristics of the operated cultures [69, 75].

A recently published study by Lee et al. illuminated that surgical mitral valve repair with lifting annuloplasty strip for patients with acute phase infective endocarditis, including HE, which sometimes has a 5-year survival. This study also showed a significant decrease in left ventricular diastolic end dimensions with no/minimal regurgitation up to a median follow-up of 54 months post operation [76]. In the past, prevention before invasive dental procedures was a model recommendation for the prevention of bacterial endocarditis of oral and pharyngeal organisms, especially HACEK organisms and streptococci of the viridans group. The recommendation was overturned by the AHA in 2007 after population-based data showed that only a few cases of IE could be prevented by prescribing prophylactic antibiotics for dental procedures [77, 78].

### Prevention and prophylaxis

Recently, prophylaxis attributable to invasive dental operations was a normal precaution for the prevention of infections endocarditis from oropharyngeal bacteria especially HACEK and viridians streptococci group. However, this suggestion was withdrawn by AHA in 2007, after population-based evidence showed that only a minimal percentage of IE cases are eliminated by the use of antibiotic prophylaxis for dental procedures [77]. Scientific studies and growing evidence continue to contradict and refine the suggested prophylactic techniques. Antibacterial prophylaxis is now approved for use in particular high-risk cases, such as the involvement of a prosthetic heart valve, prior history of IE, uncorrected or newly corrected CHD, and the occurrence of prosthetic heart valve disorders [73]. For patients in these high-risk groups, prophylaxis is recommended prior to dental operations involving stimulation of the gingival tissue or For patients in these high-risk groups, prophylaxis is recommended prior to dental operations involving stimulation of the gingival tissue or periapical area of the teeth or destruction of oral mucosa such as root canal processes [77].Antibiotic prophylaxis is not approved for using local anesthetic of non—infected areas, for the prevention of superficial caries, for the insertion of sutures, dental x-rays, for the positioning or modification of removable implantation of prosthesis or orthodontics, or for damage to the lips and mucous membranes [73].The key focus for antibiotic prophylaxis in these groups is oral streptococci, although HACEK group are also considered. The most prescribed prophylaxis are amoxicillin or ampicillin, 2 g orally or intravenously in adults and 50 mg/kg orally or intravenously in non-adults, according to the ESC 2015 standards. In the penicillin or ampicillin allergic patient, clindamycin can be chosen to give at 600 mg for adults and 20 mg/kg in in non-adults. The antibiotics are supplied as a single dose 30–60 min prior to the dental procedure [73]. However, while antibacterial prophylaxis is limited to high-risk populations, such prevention steps should be extended to all cardiac disease. The ESC Standards recommend non-specific preventive steps, such as stringent oral and skin care, 2-yearly dental follow-up, wound disinfection, therapeutic antibiotics for bacterial infection, no antibiotic self-medication and minimal use of catheters and aggressive methods [73].

## Conclusion

The epidemiology and management of infectious endocarditis treatment is constantly changing and these statistics have been reported in different countries. The guidelines provide specific recommendations for the management of infectious endocarditis. However, when making decisions about infectious endocarditis treatment, care must be taken with the individual characteristics of the patient, the type of pathogen, and the risk of disease consequences in different population and age groups.

## Data Availability

Any additional information can be obtained from the corresponding author on request.

## Abbreviations

IE: Infective endocarditis
RHD: Rheumatic heart disease
CHD: Coronary heart disease
HACEK: Haemophilus, Aggregatibacter, Cardiobacterium, Eikenella, Kingella
TTE: Transthoracic echocardiography
TEE: Transesophageal echocardiography
PPV: Positive predictive value
AHA: American heart association
MALDI-TOF MS: Laser ionization by matrix-time flight mass spectrometry
